# Comparison between two race/skin color classifications in relation to health-related outcomes in Brazil

**DOI:** 10.1186/1475-9276-10-35

**Published:** 2011-08-25

**Authors:** Claudia Travassos, Josué Laguardia, Priscilla M Marques, Jurema C Mota, Celia L Szwarcwald

**Affiliations:** 1 Instituto de Comunicação e Informação Científica e Tecnológica em Saúde (ICICT/FIOCRUZ), Avenida Brasil 4365, Manguinhos, Rio de Janeiro/RJ, CEP: 21040-900, Brazil; 2 Escola Nacional de Saúde Pública Sérgio Arouca (ENSP/FIOCRUZ), Rua Leopoldo Bulhões, 1480, Manguinhos, Rio de Janeiro/RJ, CEP: 21041-210, Brazil

**Keywords:** classification, race, skin color, health, health care, utilization, discrimination

## Abstract

**Background:**

This paper aims to compare the classification of race/skin color based on the discrete categories used by the Demographic Census of the Brazilian Institute of Geography and Statistics (IBGE) and a skin color scale with values ranging from 1 (lighter skin) to 10 (darker skin), examining whether choosing one alternative or the other can influence measures of self-evaluation of health status, health care service utilization and discrimination in the health services.

**Methods:**

This is a cross-sectional study based on data from the World Health Survey carried out in Brazil in 2003 with a sample of 5000 individuals older than 18 years. Similarities between the two classifications were evaluated by means of correspondence analysis. The effect of the two classifications on health outcomes was tested through logistic regression models for each sex, using age, educational level and ownership of consumer goods as covariables.

**Results:**

Both measures of race/skin color represent the same race/skin color construct. The results show a tendency among Brazilians to classify their skin color in shades closer to the center of the color gradient. Women tend to classify their race/skin color as a little lighter than men in the skin color scale, an effect not observed when IBGE categories are used. With regard to health and health care utilization, race/skin color was not relevant in explaining any of them, regardless of the race/skin color classification. Lack of money and social class were the most prevalent reasons for discrimination in healthcare reported in the survey, suggesting that in Brazil the discussion about discrimination in the health care must not be restricted to racial discrimination and should also consider class-based discrimination. The study shows that the differences of the two classifications of race/skin color are small. However, the interval scale measure appeared to increase the freedom of choice of the respondent.

## Introduction

During the 20^th ^century in Brazil, the discussion on race-related issues - either social, economic, educational and/or political ones - was restricted to certain fields of study, such as sociology and social anthropology. More recently, the debate has spread towards public health [1,2], and various studies in this area deal with race as a determining factor for health inequity. Such studies include race and/or skin color as one of their variables, but the complexity surrounding racial classification influences and, therefore, hampers the analysis of race as a variable, especially when it is considered as a social construct instead of a biogenetic entity [2].

The significance of phenotypic distinctions in racial classifications is related to the importance given to race as a basis for the social differentiation and stratification [3]. Besides, in racialized social systems the placement of people in racial categories involves some form of hierarchy that produces definite social relations between races [4].

Opposed to the North-American bipolarity, Brazilians identify their skin color in a multiple mode, which is less categorical and more contextual [5]. It is the product of a complex equation involving physical traits, socio-economic origin and region of residence that may result in a set of categories spread through a light-dark continuum. As shown by Sansone [5], Brazilian skin color terminology varies according to strategies individuals use to manage racial relations in several contexts (work, family, leisure, friendships) and also according to their age, education, and income.

The official classification of race/skin color in Brazil is composed by five categories - White [*Branco*], Brown [*Pardo*], Black [*Preto*], Yellow and Indigenous. Despite the controversies, some scholars have argued in favor of this classification: it refers to an objective, precise, "demographic" characteristic more suitable for census purposes than other measures that they consider more related to color identity [6]. However, the way Brazilians identify their race/skin color may fit better in a classification spread through a light-dark continuum.

The fact that measures of human skin color are based on subjective categories that vary according to the situation and over time [7], associated with limited knowledge about the genetic basis of normal variation in pigmentation within and among human population [8], brings the interpretation of the race/color variable in health studies into question. Therefore, the analysis of health studies using race/skin color as a variable must take into consideration the classification used in the study, the way this piece of information was collected (self-classification or interviewer-classification) and the types of control against possible confounding effects [1,9].

This article presents a comparison between the race/skin color classification adopted by the Brazilian Institute of Geography and Statistics (*Instituto Brasileiro de Geografia e Estatística - IBGE*) and a skin color scale [10], examining the correspondence between these two measures and exploring whether either of them is associated with health status, utilization of health services and discrimination in the health services. We assume that the skin color scale is a better measure of the social construct race/skin color than the IBGE classification, since the options it presents to the Brazilians gives more flexibility to express the way they see themselves in this regard.

## Materials and methods

This is a cross-sectional study with data obtained from the World Health Survey (WHS), a national population study that was part of a project designed by the World Health Organization (WHO) to evaluate the performance of the healthcare systems of member states [11]. In Brazil, the WHS was conducted in 2003 using a sample of 5000 individuals older than 18 years who answered a questionnaire originally designed by the WHO and adapted to the country. The sampling process was conducted in three stages: in the first stage, 250 census tracts with probability proportional to size were selected, excluding special units such as prisons, nursing homes, military bases and Indian reservations. These census tracts, called primary selection units, were explicitly stratified according to the municipality's situation (urban or rural) and size (up to 50,000; from 50,000 to 400,000; and more than 400,000 inhabitants). The average income of the heads of households in each tract was used for implicit stratification. In the second stage, 20 households in each tract were selected by inverse sampling and, in each household, one resident older than 18 years was randomly selected to respond to the individual questionnaire. One resident in each household was chosen to answer questions about the household's characteristics, resources and expenses [11].

The analysis of the race/skin color variable was performed by comparing two measures of self-classification. The first is the one adopted by the Demographic Census conducted by IBGE, which classifies individuals as white, black, brown [*pardo*], yellow, and indigenous. The second is an adaptation of a skin color scale [10] with scores that range from 1 (lightest) to 10 (darkest). Individuals who self-classified according to IBGE categories as yellow (94) or indigenous (93) were excluded from this study, due to their low frequency. Individuals who chose not to classify themselves according to IBGE categories or to the skin color scale (86) were also excluded.

The general health status of subjects was assessed through self-evaluation by means of the question: "*In general, how do you evaluate your health status?*". The options "very good" and "good" were grouped together in a category named "**good"**, and the options "moderate", "bad" and "very bad" were grouped in a category named "**bad**". In order to measure the utilization of health services, the question "*In the last 12 months, have you consulted a doctor?*" was used. Discrimination in health care was measured by means of the question *"In the last 12 months, have you thought that health professionals treated you worse than other people for any of the following reasons?" *with the following categories - lack of money, social class, sex, age, sexual orientation, or type of disease. The respondent could answer *yes *or *no *to each item, attributing her/his experience of discrimination in the health services to more than one reason.

Descriptive analysis of the two race/skin color measures was performed according to demographic characteristics (sex and age) and social characteristics (educational level, measured in years of schooling, and number of consumer goods in the household - television, fridge, sound system, telephone landline, washing machine, mobile phone, car, microwave oven, computer, and dishwasher). The weight attributed to the presence of each household good was defined as a complement of the relative frequency of each item in the total sample, that is, the rarer the item, the greater the weight attributed to it [12]. The number of goods is given as the total sum of the number of each type of good multiplied by its specific weight [13].

The similarities between the two color classifications were evaluated by means of correspondence analysis, an exploratory method for graphic representation of the multidimensional relations among the categories of the variables being studied according to the χ2 distances between them. Symmetric projection was used, making possible the analysis of the relations between lines and columns of the contingency table simultaneously, that is, the relations between all the categories of both variables under consideration. Categories located near each other on the map are more closely associated than those which are separated by greater distances. Any category, represented by a point on the map, may be analyzed separately and characterized according to its distance with respect to the projection of points representing all the other categories on a straight line that connects its plotted point to the origin of the axis on the map. Categories of the same variable situated near each other on a correspondence analysis map indicates that, irrespective of their semantic content, they may be considered equivalent with respect to the distribution of masses of the total number of observations made.

The effect of the two classifications of race/skin color in the self-assessment of health status, health care service utilization and discrimination in inpatient and outpatient health services was tested through logistic regression models for each sex, using age, educational level and ownership of consumer goods as covariables. The stratification of the multivariate analyses by sex was due to the fact that in the bivariate analysis there was a statistically significant difference between skin color scale and sex. The comparisons used a significance level of 5% using the Wald test and the values contained within confidence intervals.

In view of the multiple stages of the sampling process, all the analyses were performed according to the sampling design and used the *Statistical Package for Social Science *(version 13.0) software.

## Results

Among the 4,728 subjects studied, 54,1% were female, mean age - 40,7 years (SD 16.2) - and median age - 38 years. Average education was 7,5 years of schooling (SD 8.9) - median 6 years - and the average number of consumer goods per household was 5 (SD 2.4) - with a median of 5 consumer goods.

With regard to race/skin color, 50.8% of individuals classified themselves as white, 35.1% as brown, and 11.1% as black, according to IBGE categories. The average score in the skin color scale was 4.5 (SD 1.9) with a median of 4, varying according to IBGE categories - 3.8 for the individuals self-classified as whites, 5.2 for browns and 6.1 for blacks.

The distribution of individuals self-classified as white, brown or black according to IBGE categories in the color scale was distinct (χ^2^: 1212.5, p <0.01). The distribution of brown individuals was more concentrated in the central values of the scale; whites were more concentrated in the left side (lighter skin color) of the scale, while individuals self-classified as blacks were more dispersed along the color scale.

**Figure 1:**
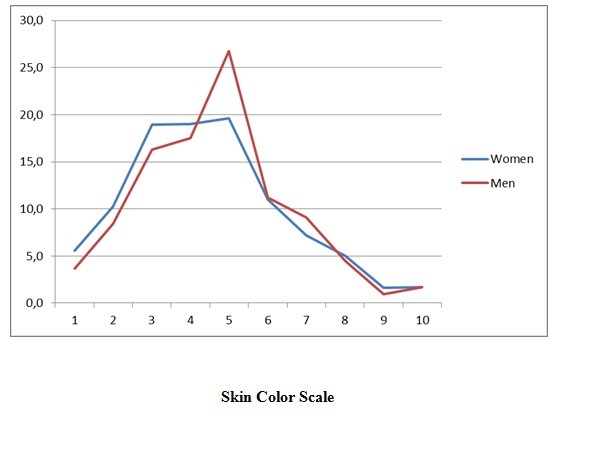
**Distribution of the skin color scale according to color (IBGE) and sex**.

Both sexes showed equal distributions along IBGE categories for race/skin color. However, the distribution of men and women in the skin color scale was different (χ^2^: 61.5, p <0.01). Distribution in the color scale according to sex showed that women tended to give themselves lower scores (lighter skin color) than men (Figure 1).

The distribution by age, schooling and consumer goods across race/skin color in each classification is presented in Table 1.

**Table 1:** Mean and standard deviation for age, years of schooling and possession of consumer goods according to racial classification and gender

Racial classification	Women			Men		
	**Age (years)**	**Years of schooling**	**Consumer goods**	**Age (years)**	**Years of schooling**	**Consumer goods**

**Race/color (IBGE)**						

*White*	42.3 (16.4)	8.1 (8.8)	5.4 (2.4)	42.8 (16.7)	8.4 (9.4)	5.5 (2.4)

*Brown*	40.5 (15.5)	6.4 (7.9)	4.1 (2.1)	41.6 (16.2)	6.4 (8.2)	4.0 (2.2)

*Black*	41.0 (16.3)	6.4 (9.5)	3.9 (2.2)	40.1 (16.1)	5.9 (7.3)	3.7 (2.1)

Total	2654	2654	2626	2074	2074	2051

**Color scale**						

*1*	45.2 (16.7)	6.3 (4.9)	5.0 (2.2)	46.9 (18.9)	6.6 (5.5)	4.7 (2.1)

*2*	42.8 (16.8)	7.4 (8.4)	5.1 (2.5)	44.3 (17.1)	7.2 (8.3)	5.0 (2.4)

*3*	41.8 (16.5)	7.8 (8.0)	5.2 (2.5)	43.6 (16.4)	7.9 (9.1)	5.2 (2.5)

*4*	41.6 (15.9)	7.7 (8.6)	4.9 (2.4)	42.6 (16.6)	7.7 (8.7)	5.1 (2.4)

*5*	40.2 (15.3)	7.2 (7.7)	4.8 (2.3)	40.6 (16.1)	7.0 (6.9)	4.8 (2.3)

*6*	38.5 (14.7)	7.5 (9.7)	4.6 (2.4)	39.7 (15.2)	8.7 (11.7)	4.6 (2.4)

*7*	40.7 (15.6)	7.1 (7.8)	4.4 (2.1)	37.9 (15.4)	6.7 (4.5)	4.3 (2.2)

*8*	41.0 (16.0)	7.1 (10.9)	3.9 (2.3)	44.2 (17.8)	5.5 (5.0)	4.0 (2.5)

*9*	43.7 (16.6)	7.0 (13.6)	4.3 (2.2)	40.1 (14.6)	4.3 (5.7)	2.8 (1.9)

*10*	42.0 (16.3)	4.7 (3.6)	3.7 (2.0)	43.3 (17.7)	4.6 (3.6)	3.7 (1.9)

**Total**	2586	2586	2560	2026	2026	2003

**Figure 2:**
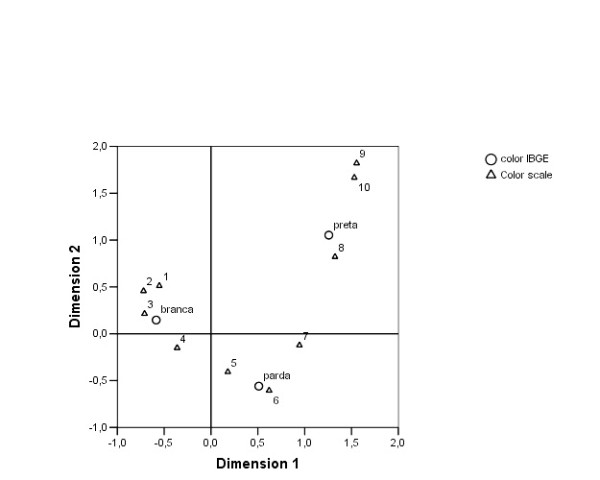
**Correspondence analysis**. Color classification according to IBGE categories and to the skin color scale. Brazil, 2003.

In the graphical representation of correspondence analysis (Figure 2), the first dimension (horizontal axis) accounts for 77% of the relationship between the two classifications, ordering the categories of both variables on a continuum that ranges from white (IBGE) and scores 1 to 3 (color scale) on the left part of the axis to black and scores 8 to 10 on the opposite side; the remaining categories occupy intermediate positions. This dimension represents the gradation of skin color of individuals, while the second dimension separates the black category and the highest (darker) scores in the color scale from the brown category and scores 4 to 7.

In the multivariate analysis of self-assessment of health status for women and men, it was observed that the chance that a person would self-assess their health status as "bad" increased with age and decreased with years of schooling and number of goods. Skin color showed no effect in any of the two measures (Table 2), and there was no significant variation between men and women.

**Table 2:** Explanatory model for self-assessment of health status according to gender and to racial classification using IBGE categories and the skin color scale

Variables	*Women*			*Men*		
***IBGE***	**OR**	**CI 95%**	**Sig**	**OR**	**CI 95%**	**Sig**

**Age**	1.04	1.03-1.04	0.00	1.03	1.03-1.04	0.00

**Skin color (IBGE)**						
White	1.00	-	-	1.00	-	-
Brown	0.84	0.69-1.03	0.95	1.01	0.79-1.29	0.96
Black	0.95	0.72-1.26	0.72	1.11	0.72-1.72	0.64

**Years of schooling**	0.91	0.90-0.94	0.00	0.93	0.90-0.96	0.00

**Consumer goods**	0.87	0.83-0.92	0.00	0.93	0.87-0.97	0.02

*Skin color scale*						

**Age**	1.03	1.03-1.04	0.00	1.03	1.03-1.04	0.00

**Skin color scale**	1.01	0.96-1.04	0.61	1.03	0.97-1.10	0.31

**Years of schooling**	0.92	0.90-0.94	0.00	0.93	0.90-0.96	0.00

**Consumer goods**	0.87	0.82-0.91	0.00	0.93	0.87-0.99	0.02

Brazil, 2003

In the bivariate analysis of the use of health services, whites (55.9%) used more services than browns (33.3%) and blacks (10.8%) (p <0.05), but the skin color scale did not show statistically significant differences. When estimating a multivariate model for women and men, the effect of skin color in the use of health services was not statistically significant in any of the two measures, except for brown women that showed a greater chance of use compared to white women (Table 3). As expected, the need for health care, measured by self-assessment of health status, was the main factor influencing the use of health services for men and women. Social inequality in the use of health services was observed, with the odds of use adjusted by need (self-assessment) and age being lower in persons with fewer consumer goods in both models - classification according to IBGE categories and the skin color scale. A higher education level also positively affected the use of health services in both classifications.

**Table 3:** Explanatory model for use of health services in the last 12 months according to gender and to racial classification using IBGE categories and the skin color scale

Variables	*Women*			*Men*		
***IBGE***	**OR**	**CI 95%**	**Sig**	**OR**	**CI 95%**	**Sig**

**Age**	1.00	0.99-1.01	0.60	1.00	0.99-1.01	0.58

**Skin color (IBGE)**						
White	1.00	-	-	1.00	-	-
Brown	1.40	1.03-1.89	0.03	0.90	0.68-1.20	0.48
Black	0.91	0.56-1.48	0.70	088	0.58-1.33	0.54

**Years of schooling**	1.06	1.01-1.10	0.01	1.05	1.02-1.09	0.00

**Consumer goods**	1.08	1.00-1.12	0.04	1.09	1.03-1.17	0.01

Self-assessment of health status*	1.56	1.14-2.14	0.01	1. 63	1.22-2.18	0.00

*Skin color scale*						

**Age**	1.00	0.99-1.01	0.71	1.00	0.99-1.01	0.59

**Skin color scale**	0.97	0.90-1.05	0.42	1.01	0.96-1.07	0.69

**Years of schooling**	1.06	1.01-1.10	0.01	1.05	1.02-1.09	0.00

**Consumer goods**	1.10	1.02-1.18	0.01	1.10	1.03-1.18	0.00

Self-assessment of health status*	1.55	1.13-2.15	0.01	1.63	1.21-2.19	0.00

Brazil, 2003

(*) Reference category: Good and very good.

The study also addressed the issue of discrimination in the healthcare services, either in outpatient or inpatient care. One must note that this analysis was based on a one single item focused on the individual experience with healthcare professionals within the previous year. Therefore, it does not intend to inform how positive responses affected the care received [14]. Among the reported reasons for discrimination, lack of money (8.6% in outpatient care and 12.5% in hospitalized patients) and social class (7.7% in outpatient care and 10.8% in hospitalized patients) were the most frequent. Skin color was the least reported reason for discrimination among the available options (1.1% in outpatient care and 1.6% in inpatients). However, it was observed that, among inpatients, persons with darker skin color reported discrimination more frequently for each of the reasons for discrimination, in both measures of race/skin color classification. A higher percentage of discrimination due to skin color was also reported by people who self-classified as black and by those with scores higher than 6 in the color scale, with frequencies of 7.0% and 5.9%, respectively. With regard to the type of service, reports of discrimination were more frequent in hospitalized patients than for ambulatory care for all forms of discrimination addressed by the study.

Bivariate analysis showed that age, years of schooling, number of consumer goods and self-classification according to IBGE categories were also related to statistically significant differences in the reports of any type of discrimination. People who felt discriminated against were younger, had between 8 and 11 years of schooling, fewer consumer goods and classified themselves as brown. The skin color scale, though not reaching statistical significance, also showed that intermediate scores reported a higher percentage of discrimination. Multivariate analysis aimed at testing whether there was an association between referring any type of discrimination during inpatient or outpatient care and race/skin color (IBGE/color scale) and number of consumer goods showed a statistically significant association for men when the skin color scale was used (OR: 1.15, CI 95%: 1.05 to 1.27) - Table 4.

**Table 4:** Explanatory model for discrimination in health care according to gender and to racial classification using IBGE categories and the skin color scale

Variables	*Women*			*Men*		
***IBGE***	**OR**	**CI 95%**	**Sig**	**OR**	**CI 95%**	**Sig**

**Age**	0,99	0,98-1,00	0,05	0,98	0,97-0,99	0,0

**Skin color (IBGE)**						
White	1,00	-		1,00		
Brown	0,89	0,67-1,18	0,41	1,02	0,68-1,54	0,92
Black	0,77	0,49-1,22	0,26	1,27	0,72-2,22	0,40

**Years of schooling**	0,96	0,92-1,00	0,05	0,97	0,92-1,02	0,21

**Consumer goods**	0,96	0,89-1,03	0,28	0,89	0,81-0,99	0,03

*Skin color scale*						

**Age**	0,99	0,98-1,00	0,44	0,98	0,97-0,99	0,00

**Skin color scale**	0,99	0,93-1,06	0,72	1,15	1,05-1,27	0,00

**Years of schooling**	0,96	0,92-1,00	0,47	0,97	0,92-1,02	0,17

**Consumer goods**	0,96	0,88-1,03	0,24	0,90	0,82-0,99	0,04

Brazil, 2003

## Discussion

The proximity between categories of the two measures of skin color observed in the correspondence analysis indicates the existence of similarities among them, pointing that both represent the same race/color construct. On the other, the distance between white, brown and black, as well as between the color scale categories, shows their distinctiveness.

Brazilians tend to self-classify their skin color near the center of the color gradient, in agreement with results from other studies that reported the preference of Brazilians for the brown [*moreno*] category and its association with the idea of a mixed-race nation [6], i.e., neither white nor black. This tendency, observed in the skin color scale, was more evident among people who self-classified as white than among those who self-classified as black according to IBGE categories. The distribution of individuals who self-classified as black along the color scale seems to indicate that, among this group, the value attributed to their skin color is less clear, or that "black" represents more a political feature adopted by some, indicating African ancestry and not necessarily the color of their skin.

An important result to be highlighted is the fact that women tended to self-classify their skin color as a little lighter than men when the color scale was used; this gender effect was not observed when IBGE categories were used. The gender effect captured by the scale may be influenced by sociocultural factors, such as the fact that the media and the sexual-affective market assign distinct values for men and women as regards the standard of beauty, so that women with lighter skin have increased chances of undergoing a sexual-affective relationship [15].

With regard to the health outcomes analyzed in the study, race/skin color was not a relevant factor to explain any of them, regardless of the race/skin color classification. The models tested here were in accordance with the Brazilian literature on the subject, which states that the effect of race/color in the self-assessment of health status and use of health services disappears after adjustment for other socioeconomic variables, one exception being brown women that were more likely to use health services compared to women in the others skin color categories. The well-known social inequality in the use of health services in Brazil [16] is not associated with race/skin color, but with education and economic status, measured by the number of consumer goods.

In the analysis of discrimination by health professionals, around 10% of subjects who used health services in the last 12 months reported some form of discrimination, and a much lower percentage reported discrimination associated with race/skin color. One aspect stand out in the multivariate analysis of discrimination in health services - only the models for men showed statistically significant associations. In such cases, younger age and a lower number of consumer goods in the household were associated with discrimination when IBGE categories were used, while younger age, lower number of goods and skin color were associated with discrimination when the color scale was used. The fact that men reported more experiences of discrimination in health services may be explained by the hypothesis that such services are much more receptive to women despite their social status, probably because they are the main users of those services [17]. The fact that younger and poorer men felt more discriminated against in the health services may be related both to the stereotype of marginalization commonly reported in the media, where police news often depict young men with signs that associate them with the poorer strata, and to a higher awareness by these young men with regard to their rights, since they are frequent targets of discriminatory attitudes in public spaces [18]. In addition, health services may be perceived by these young men as unfamiliar environments.

The results of this study suggest that in Brazil the discussion about discrimination in the health services must not be restricted to racial discrimination and should also consider class-based discrimination. In order to improve the quality of health care, one must take into account that health professionals, particularly doctors, are not immune to prejudice and stereotyping when making decisions about their patients. In Brazil, socioeconomic status seems to stand out as the main feature for discrimination in health, despite the fact that this is not a pattern observed elsewhere [19].

With regard to the limitations of this study, some aspects are worth mentioning. The first is the sample size for respondents who self-classified as blacks, given that the sample design aimed to make the sample representative for the Brazilian population over 18 years of age, regardless of skin color classification. Although the number of respondents who self-classified as blacks (10.5%) was close to the values found in surveys made by IBGE, the small number of participants may lead to less accurate estimates. As the measures of skin color vary: one is ordinal and the other numerical statistical analyses of the latter have more power to detect statistically significant association than the former, given that it contains more information. Despite the interaction effects of schooling and possession of consumer goods mentioned in the literature, the inclusion of these criteria in the models did not result in a significant improvement of estimates; for that reason, we chose to work with simpler models.

The two measures used in this study - a color scale and categorical variables - stress the marked distinction between the semantic field of a word (white), which underlies the entire sociohistorical usage of the word, and a number in a continuum. As the concept of race is a social construct, the categories in its classification bring with them the dominant ideological values established in society. The differences between the two classifications of race/skin color, despite being small, suggest that an interval scale increases the freedom of choice of the respondent for, apart from containing more options, it is not based on discrete, historically-constructed categories.

## Final Thoughts

The ambiguity attributed to racial classification in Brazil is associated with miscegenation, because people do not fit clearly into one category or another, as such categories are based on popular stereotypes rather than precise legal definitions [20]. That ambiguity can be seen as an evidence that Brazilians are using more than one way of classifying race, including those classifications that comprise intermediary possibilities. The fact that Brazilians may fit into more than one group according to the context and the situation being experienced would favor the use of a classification that takes this ambiguity into account. Linguistic categorization itself, taken as a cognitive phenomenon, postulates the vagueness of categorical boundaries [21] - in contrast with the classical view of categories as well-defined entities - and the different levels of representation of the elements that constitute each category [22], i.e., the existence of more or less prototypical elements. According to Schwartzman [23], the fluidity, imprecision, and variation among generations observed in the issue of racial classification in Brazil point to the adoption, by administrative agencies, of criteria that take into account the cultural and social permeability observed in the country. If this, on one hand, puts in jeopardy the objectivity of color attribution, on the other hand, it serves to show significant differences in Brazilian culture [24].

## Competing interests

The authors declare that they have no competing interests.

## Authors' contributions

CT and JL conceived the study, performed the data analysis and drafted the manuscript. PM participated of the discussion, drafted the manuscript and revised it. JCM performed the statistical analysis. CL participated in the conception and development of the World Health Survey, and revised the statistical analysis. All authors read and approved the final manuscript.
